# Hydrochlorothiazide combined with exercise training attenuates blood pressure variability and renal dysfunctions in an experimental model of hypertension and ovarian hormone deprivation

**DOI:** 10.1096/fba.2024-00168

**Published:** 2025-03-04

**Authors:** Pietra Petrica Neves, Maycon Junior Ferreira, Tânia Plens Shecaira, Marina Rascio Henriques Dutra, Débora Conte Kimura, Guiomar Nascimento Gomes, Kátia De Angelis

**Affiliations:** ^1^ Laboratory of Translational Physiology Nove de Julho University (UNINOVE) São Paulo São Paulo Brazil; ^2^ Department of Physiology Federal University of São Paulo (UNIFESP) São Paulo São Paulo Brazil

**Keywords:** blood pressure variability, exercise training, hydrochlorothiazide, hypertension, kidney

## Abstract

Considering that blood pressure variability (BPV) has been associated with damage to target organs such as the kidneys, this study aimed to investigate the effects of the association of hydrochlorothiazide (HCTZ) combined with exercise training (CET) on BPV, as well as morphology, function, inflammation, and oxidative stress in renal tissue. Our study was designed to experimentally simulate arterial hypertension associated with the postmenopausal period in females, a condition linked to a considerable increase in cardiovascular risk. To replicate the physiological cessation of ovarian hormones, we performed a bilateral ovariectomy. Female spontaneously hypertensive rats (SHR) were distributed into 4 ovariectomized groups (*n* = 5–6/group): sedentary (OS), sedentary + HCTZ (OSH), trained (OT), and trained + HCTZ (OTH). Both HCTZ (3 mg/kg) and CET (3 days/week) were performed for 8 weeks. Blood pressure (BP) was directly recorded for BPV analyses. Renal function, morphology, inflammation, and oxidative stress were evaluated. The OSH, OT, and OTH groups had lower systolic BP (SBP) (OSH: 189 ± 13; OT: 179 ± 5; OTH: 174 ± 15 mmHg) when compared to the OS group (208 ± 15 mmHg). Only the association of the drug with CET promoted a reduction in variance of SBP. The groups treated with HCTZ showed lower plasma creatinine levels and increased creatinine clearance compared to the OS group. Treated groups showed a reduction in fields of 51%–100% of interstitial tubule fibrosis when compared to the OS group, and the OTH group also showed reduction in fields in the range of 26%–50% versus other groups. There was an increase in renal catalase, a reduction in IL‐6, and an increase in IL‐10 in the OTH group. Positive correlations were obtained between variance of SBP and SBP (*r* = 0.72), plasma creatinine (*r* = 0.58), IL‐6 (*r* = 0.62), hydrogen peroxide (*r* = 0.61), and protein oxidation (*r* = 0.66), as well as between vascular sympathetic modulation and lipoperoxidation (*r* = 0.62) in kidney tissue. In conclusion, our findings highlight the enhanced effectiveness of combining HCTZ and CET compared to using the drug alone in the studied model. This dual approach may provide additional cardiovascular and renal benefits beyond reduction of BP, potentially leading to improved quality of life and reduced morbidity associated with systemic arterial hypertension.

## Graphical abstract

Hydrochlorothiazide plus combined exercise training provides superior benefits compared to medication alone in female spontaneously hypertensive rats subjected to ovarian hormone deprivation. This combined approach significantly reduced blood pressure variability, improved inflammatory and redox profiles, and provided functional and morphological benefits to renal tissue. From a translational perspective, replicating similar results in humans would underscore the fundamental importance of incorporating regular exercise training into antihypertensive pharmacological treatment, providing additional benefits beyond blood pressure reduction, potentially reducing hypertension‐related complications.

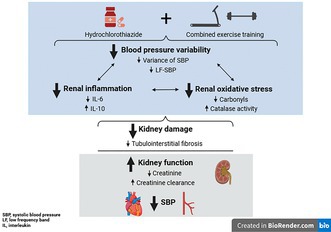

## 1 INTRODUCTION

Systemic arterial hypertension (SAH) is currently one of the main causes of morbidity and mortality in the world, leading to the development of several cardiovascular diseases (CVDs) and lesions in target organs, such as the kidney.^1^, ^2^, ^3^ SAH is related to chronic kidney disease (CKD) and is currently one of the main causes of this disease. The coexistence of these two diseases significantly exacerbates cardiovascular risk, thereby leading to a substantial increase in mortality corresponding to the severity of CKD.^4^ In addition, females are susceptible to a higher risk of CVDs due to the aging process, with a high cardiovascular risk and increased prevalence of SAH and CKD after menopause.^5^

Studies demonstrate that reducing blood pressure (BP) in hypertensive patients reduces the risk of cardiovascular outcomes.^6^ Importantly, in addition to absolute BP values, increased BP variability (BPV) is associated with the development, progression, and severity of cardiac, renal, and vascular damage, increasing the risk of cardiovascular mortality,^3^ suggesting as a potential marker of damage in target organs. In addition, it has been knowledge that the autonomic nervous system can modulate inflammation, which suggests that the reduction in heart rate variability or even the increase in BPV may be associated with an increase in the release of pro‐inflammatory cytokines and, consequently, an increase in oxidative stress^7^ which could favor damage to target organs such as the kidneys.

Pharmacological and non‐pharmacological therapies are classically adopted to treat SAH. In fact, pharmacological treatment is one of the main measures, with proven effectiveness in reducing BP, which can be obtained with the continuum use of different drug classes. Thiazide diuretics, among which is hydrochlorothiazide (HCTZ), constitute one of the preferable drug classes prescribed in the treatment of SAH due to its therapeutic efficacy and its low cost.^8^ However, current knowledge suggests a conflicting effect of HCTZ on the mechanisms involved in the pathogenesis and progression of SAH.^9^, ^10^, ^11^ Such evidence leads to the understanding that a potential risk still remains even when there is antihypertensive medication treatment and BP control. On the other hand, exercise training has also been recommended in the management of SAH, as a conservative measure to promote a reduction in BP and CKD.^12^, ^13^ Notably, exercise training can promote a decrease in the progression of renal dysfunction and outcomes, in addition to improving health when associated with another intervention.^14^, ^15^, ^16^

Our group has demonstrated the advantages of combining aerobic plus resistance exercise training with drug therapies.^17^, ^18^ However, there is no solid evidence regarding the potential benefits of combining both approaches particularly concerning kidney health. Therefore, the objective of this study was to assess the impact of combined exercise training (CET) associated with HCTZ treatment on BPV, an important marker of target organ damage, as well as on renal tissue morphology, function, inflammation, and oxidative stress in an experimental model of SAH and ovarian hormone deprivation. The limited effectiveness in managing the disease in the female population,^1^ coupled with the various physiological changes resulting from ovarian hormone deprivation, highlights the need for investigations that evaluate therapies potentially more effective not only in managing the disease during a transitional physiological period of a woman's life but also in reducing complications associated with disease progression. Additionally, our study contributes to ongoing efforts to investigate the female sex, which is underrepresented and often subject to approaches based on research conducted in males.

## 2 MATERIALS AND METHODS

Twenty‐eight female spontaneously hypertensive rats (SHR, *rattus norvegicus*) (150–200 g, 90 days old) were allocated into the following 4 groups (*n* = 5‐6/group): ovariectomized sedentary (OS), ovariectomized sedentary treated with HCTZ (OSH), ovariectomized trained (OT), and ovariectomized trained treated with HCTZ (OTH). They received standard laboratory chow and water ad libitum and were housed in cages (two to four animals) in a temperature‐controlled room (22°C–25°C) under a 12‐h dark/light cycle. All rats were treated in a similar manner in terms of daily manipulation. Based on Three Rs principles in animal experimentation, we reused some data from groups previously published^19^ for this new investigation. The research protocol was recorded under number 7611290618 and approved by the Committee for Ethics in the Use of Animals (CEUA)—Federal University of Sao Paulo (UNIFESP).

### 2.1 Ovariectomy

The ovariectomy of the animals was performed at 3 months of age. For anesthesia, initially a dose of meloxicam (1 mg/kg, 30 min before surgery) was administered subcutaneously. Additionally, ketamine (80 mg/kg) and xylazine (12 mg/kg) were administered intraperitoneally. In surgery, the oviduct was sectioned, and the ovaries removed.^20^ The animals received an intramuscular dose of penicillin (10,000 U/kg). After the procedure, a subcutaneous dose of meloxicam (1 mg/kg) was administered every 24 h and tramadol (20 mg/kg) every 8 h for 3 days for analgesic effects.

### 2.2 Pharmacological treatment

HCTZ (30 mg/kg) was administered orally (diluted in drinking water) for 8 weeks. The choice of HCTZ as a drug treatment was based on its proven effectiveness in reducing cardiovascular mortality, as well as BP, with wide acceptance and a high degree of recommendation in clinical practice.^2^

### 2.3 Combined exercise training

Maximum tests on a treadmill and an adapted ladder for rats were conducted to assess exercise capacity and prescribe CET for the OT and OTH groups, as previously described.^17^ CET was performed over 8 weeks, with sessions lasting 40–60 min at progressively increasing intensity, held three times per week. The sessions started with aerobic exercise training (40%–60% maximal exercise test velocity, 30–40 min) followed by resistance exercise training (15–20 climbs on adapted stairs per session, with 1 min of interval).^17^

### 2.4 Assessment of kidney function

For evaluation of renal function, the rats were kept for 24 h in metabolic cages (Nalgene, Ugo Basile, Italy) at the end of the seventh week. The urine sample was stored in a freezer at −80°C for later measurement of creatinine and urea levels. Plasma and urine creatinine levels were measured by the Jaffé method.^21^ Plasma urea accumulation was determined by an enzymatic colorimetric method using a specific kit (Labtest Diagnostics, Lagoa Santa, Brazil). Urinary protein excretion followed the method recommended by Bradford.^22^

### 2.5 Hemodynamic assessment

After 8 weeks of control or intervention (pharmacological treatment and/or CET), the animals received an intraperitoneal dose of ketamine (80 mg/kg) and xylazine (12 mg/kg) as anesthesia and were placed in dorsal decubitus to perform a small incision close to the neck for implantation of a cannula in the carotid artery towards the left ventricle for direct recording of BP and in the jugular vein for infusion of drugs. After that, the larger caliber ends of the cannulas were passed subcutaneously, exteriorized on the back of the cervical region, and fixed with cotton thread on the skin.^17^, ^20^ The cannulas were made with polyvinyl chloride tubes (Abbott) equivalent to polyethylene PE10 and PE50. Subcutaneous doses of tramadol (5 mg/kg) and dipyrone (50 mg/kg) were administered for analgesia (every 12 h).

Hemodynamic measurements were performed in conscious and awake rats in their cages for at least 24 h after catheter placement. All animals were housed in individual cages and subjected to the same conditions regarding HCTZ availability and duration of HCTZ exposure before and after hemodynamic assessments until euthanasia. The arterial cannula was connected to a transducer (Blood Pressure XDCR, Kent© Scientific, Litchfield, CT, USA), and BP signals were recorded over a 30‐min period using a microcomputer equipped with an analog‐to‐digital converter (Windaq, 2‐kHz sampling frequency; Dataq Instruments). The recorded data were analyzed on a beat‐to‐beat basis to quantify changes in systolic BP (SBP).^17^, ^18^

### 2.6 Blood pressure variability

BPV was analyzed in the time and frequency domains using CardioSeries software (version 2.4, CardioSeries Software, Sao Paulo University, Brazil). The entire tachogram was visualized by plotting the pulse interval over time, and the three most stable five‐minute uninterrupted sequences from the total recording period were selected: one sequence at the beginning, one in the middle, and one at the end of the 30‐minute BP recording. Each sequence was analyzed individually for BPV, and the mean values of the three sequences were calculated for each animal. The time‐domain and frequency‐domain analyses included calculating the variance of SBP and the low‐frequency component of SBP (LF‐SBP), respectively. In the spectral power analysis, the low‐frequency band with a range of 0.20–0.75 Hz was considered.

### 2.7 Tissue collection

On the day following the hemodynamic evaluations, the rats were anesthetized with ketamine and immediately euthanized by decapitation. The kidney was removed immediately after euthanasia and frozen at −80°C for analyses. Blood samples were collected, centrifuged to separate the blood components and frozen at −80°C.

### 2.8 Kidney histology

After washing in saline solution, the right kidney (5 animals per group) was kept in Bouin's solution for 24 h. After the fixation period, the pieces were dehydrated, cleared, and paraffined following the methodology recommended by Michalany.^23^ Histological sections approximately 5 μm thick were stained using Masson's trichrome technique for analysis of general morphology, such as damage tissue and glomerular quantification (mean of 40 fields per group).

Image capture was carried out with the aid of a computerized system, consisting of a polarized light microscope (Carl Zeiss) adapted to a high‐resolution camera connected to a computer. Area and glomerular count were determined by computerized morphometry (Nikon, NIS‐Elements), with 20 fields being analyzed on each slide (20× magnification). The percentage of the area with morphological alterations in the renal interstice was estimated with the aid of ImageJ software (NIH).^24^ In the tubulointerstitial morphological analysis, the fields were divided into three ranges of morphological alteration: 0%–25%, 26%–50%, and 51%–100% of the evaluated field.

### 2.9 Inflammatory mediators

TNF‐α and interleukin 6 (IL‐6) and 10 (IL‐10) levels were measure using the ELISA method in microplates (96 wells) sensitized with the antibody for the protein of interest, adhered to the wall of the plate wells by an immune adsorbent substrate. It was performed the prior blocking of nonspecific bindings and subsequent incubation of experimental samples, containing the protein (antigen) to be measured. Incubation with antibody linked to enzyme secondary antibody conjugated to peroxidase was performed, and subsequent reaction with chromogen. On the same plate, we performed the standard curve, which was used to calculate the values of amount of protein per well. The absorbance was measured in an ELISA reader device at 450 and 550 nm using specific kits for rats (R&D Systems).

### 2.10 Oxidative stress

The kidneys were homogenized for 30 s in an Ultra‐Turrax homogenizer, with 1.15% KCl and phenyl methyl sulfonyl fluoride (PMSF), at a concentration of 100 mmol/L in isopropanol and in the amount of 10 μL/mL of added KCl. They were then homogenized, centrifuged for 10 min at 3000 rpm, in a refrigerated centrifuge between 0 and 4°C and the supernatant frozen in a freezer at −80°C for dosages in later analyses.

### 2.11 NADPH oxidase

The activity of the NADPH oxidase enzyme was evaluated with renal tissue homogenate and evaluated by the production of superoxide determined by means of ELISA. To perform the assay, a 50 mM phosphate buffer containing 2 mM EDTA, 150 mM sucrose, 1.3 mM NADPH, and 10 μL of renal tissue sample was used.^25^

### 2.12 Hydrogen peroxide dosage

To occur the reaction, aliquots of 70 μL of renal tissue homogenate were incubated for 30 min in a solution containing dextrose buffer, 0.28 mmol/L of phenol red, and 5 mg of HRP. After this incubation period, 0.5 mol/L of NaOH was added, and the absorbance values of the solution measured at 610 nm. The results were expressed in μM.^26^

### 2.13 Catalase (CAT)

To carry out the CAT measurements, we used a buffer solution consisting of phosphates at 50 mmol/L at pH 7.4. Then, 980 μL of this buffer and 10 μL of renal tissue homogenate sample were added to the spectrophotometer cuvette, and this mixture was discounted against a phosphate buffer blank. Next, 10 μL of hydrogen peroxide (0.3 mol/L) was added, and the decrease in absorbance at 240 nm was monitored in the spectrophotometer.^27^

### 2.14 Superoxide dismutase (SOD)

SOD activity was determined by measuring the velocity of formation of oxidized pyrogallol. In the reaction medium, 5 μL of renal tissue homogenate, 980 μL of Tris‐Phosphate buffer at 50 mmol/L (pH 8.2), 10 μL of pyrogallol at 24 mmol/L, 5 μL of CAT at 30 μmol/L were used. This curve obtained is used as a blank. A standard curve is also performed using two different concentrations of 21 SOD (0.72 U and 0.144 U); the equation of the straight line was obtained to perform the calculations.^28^

### 2.15 Thiobarbituric acid reactive substances (TBARS)

To occur the reaction, 0.25 mL of homogenate was added to 0.75 mL of trichloroacetic acid (TCA) at 10% (W/V), which has the function of denaturing the proteins present and acidifying the reaction medium. This mixture was stirred and centrifuged for 3 min at 1000 rpm. 0.5 mL of the supernatant was removed, and 0.5 mL of thiobarbituric acid (TBA) 0.67% (W/V) was added, which reacted with the lipoperoxidation products forming a pink‐colored compound. The mixture was incubated for 15 min at 100°C and then cooled on ice. Then, the absorbance reading at 535 nm was performed in a spectrophotometer.^29^

### 2.16 Protein oxidation (Carbonyls)

The technique is based on the reaction of protein oxidation in renal tissue with 2.4 dinitrophenyl hydrazine (DNPH) in acid medium, followed by successive washings with acids and organic solvents and final incubation with guanidine.^30^

### 2.17 Statistical analysis

The collected data are presented as mean ± standard deviation, and their distributions and homogeneity were tested using the Shapiro–Wilk and Levene test, respectively. In addition, the two‐way analysis of variance (ANOVA) was used, followed by the Student–Newman–Keuls post hoc test. The relationship between parameters was analysed by Pearson correlation analysis. The adopted significance level was *p* < 0.05.

## 3 RESULTS

### 3.1 Body weight and functional capacity

In this investigation, there was no difference in body weight between the studied groups at the end of the protocol (OS: 238 ± 6; OSH: 230 ± 9; OT: 233 ± 7; OTH: 220 ± 9 g).

**FIGURE 1:**
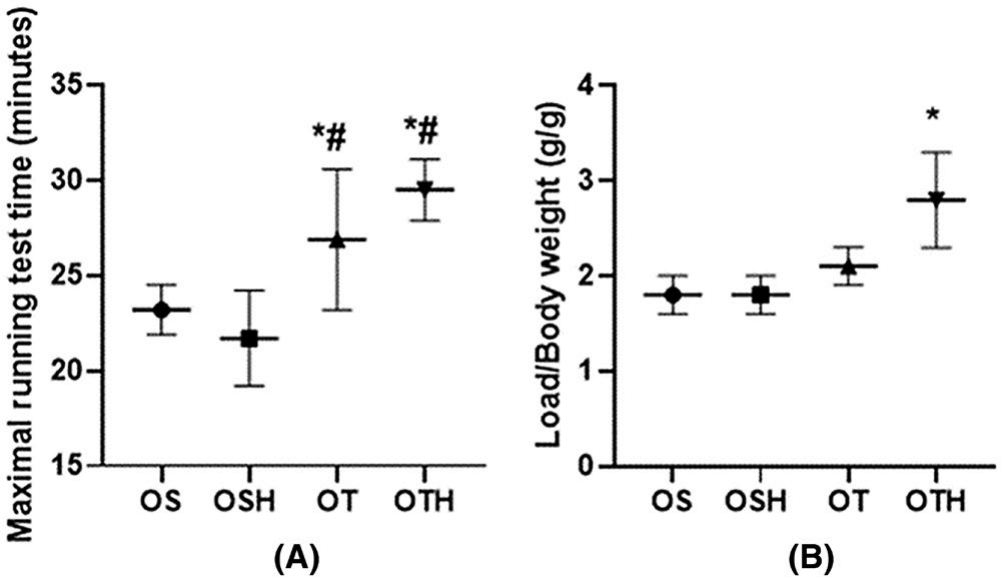
(A) Maximum time (min) and (B) maximum load/body weight (g/g) in exercise test in the studied groups. OS, ovariectomized sedentary; OSH, ovariectomized sedentary treated with hydrochlorothiazide; OT, ovariectomized trained; OTH, ovariectomized trained treated with hydrochlorothiazide. *p < 0.05 versus OS; ^#^ *p* < 0.05 versus OSH.

Both trained groups (OT and OTH) showed an increase in their performance on the treadmill (OS: 23.2 ± 1.3; OSH: 21.7 ± 2.5; OT: 26.9 ± 3.7; OTH: 29.5 ± 1.6 min). In addition, the OTH group showed an additional increase in performance in the adapted ladder test when compared to the OT group at the end of the protocol (OS: 1.8 ± 0.2; OSH: 1.8 ± 0.2; OT: 2.1 ± 0.2 vs. OTH: 2.8 ± 0.5 g) (Figure 1A,B).

### 3.2 Hemodynamic and BPV assessments

**FIGURE 2:**
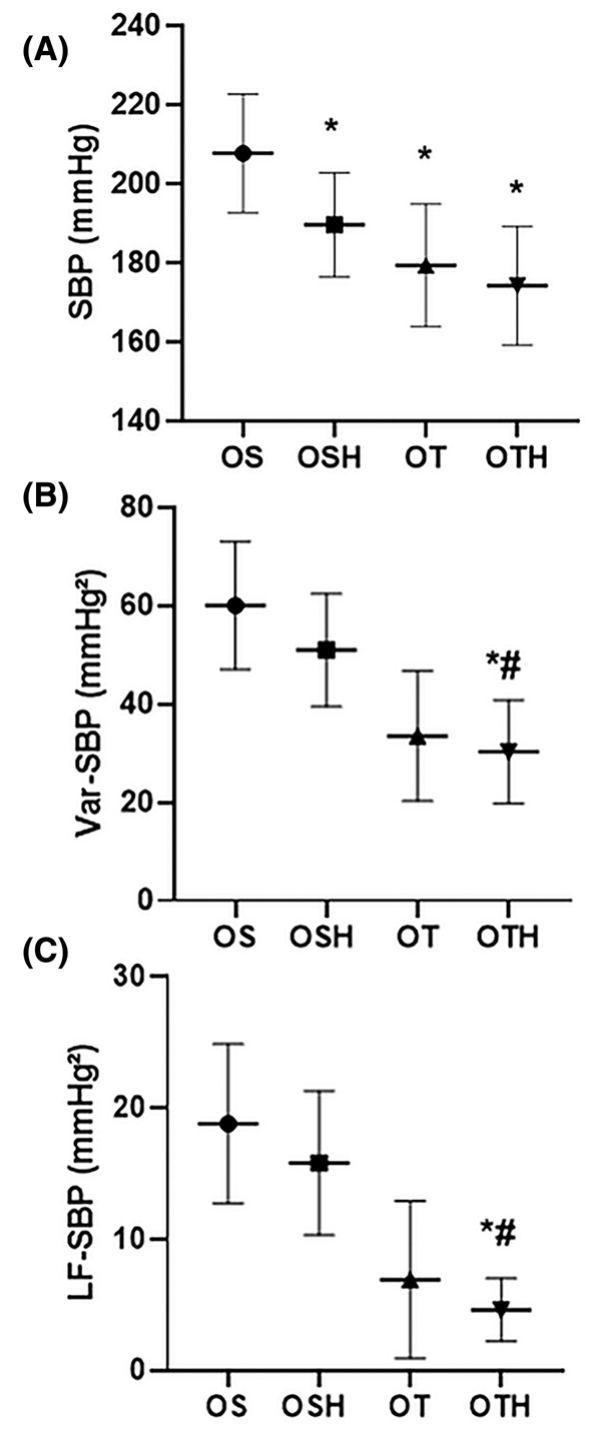
Hemodynamic and autonomic assessments in the studied groups. (A) Systolic blood pressure; (B) Variance of systolic blood pressure (Var‐SBP); (C) Low frequency band of systolic blood pressure (LF‐SBP). OS, ovariectomized sedentary; OSH, ovariectomized sedentary treated with hydrochlorothiazide; OT, ovariectomized trained; OTH, ovariectomized trained treated with hydrochlorothiazide. *p < 0.05 versus OS; ^#^ *p* < 0.05 versus OSH.

Regarding hemodynamic parameters, the sedentary group treated with HCTZ (OSH: 189.5 ± 13.2 mmHg) and the trained groups (OT: 179.3 ± 15.5 and OTH: 174.1 ± 15 mmHg) presented lower SBP values when compared to the ovariectomized sedentary group (OS: 207.6 ± 15 mmHg) (Figure 2A). In addition, the combination of therapies (OTH group) promoted a reduction in variance of SBP (OS: 60.1 ± 13 and OSH: 51.0 ± 11.5 vs. OTH: 30.3 ± 10.5 mmHg^2^) and in vascular sympathetic modulation (LF‐SBP) (OS: 18.8 ± 6.1 and OSH: 15.8 ± 5.5 vs. OTH: 4.6 ± 2.4 mmHg^2^) compared to the sedentary groups. However, any differences were observed in relation to OT group for both parameters (OT: 33.5 ± 13.2 and 6.9 ± 6.0, respectively) (Figure 2B,C).

### 3.3 Kidney function

**FIGURE 3:**
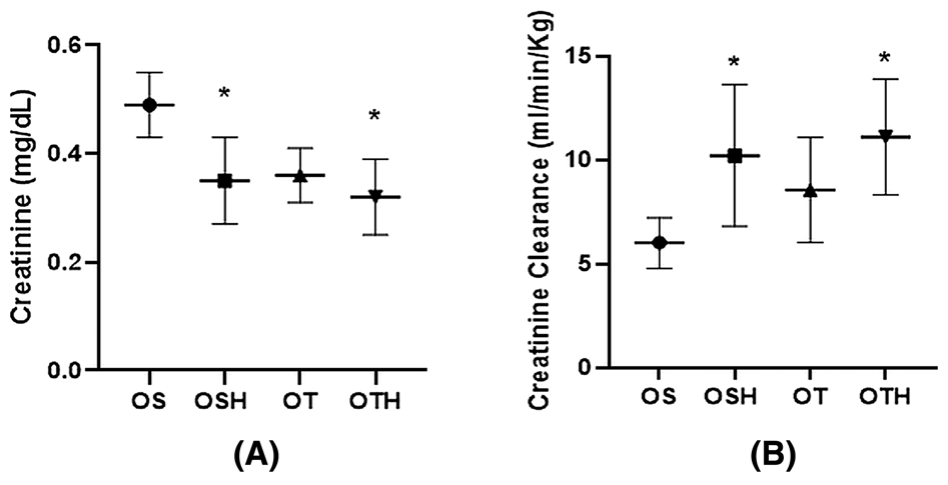
Renal function parameters in the studied groups. (A) Plasma creatinine; (B) Creatinine clearance. OS, ovariectomized sedentary; OSH, ovariectomized sedentary treated with hydrochlorothiazide; OT, ovariectomized trained; OTH, ovariectomized trained treated with hydrochlorothiazide. *p < 0.05 versus OS.

In the evaluations of renal function, the groups treated with HCTZ (OSH and OTH) showed a reduction in plasma creatinine (OS: 0.49 ± 0.06; OSH: 0.35 ± 0.08; OT: 0.36 ± 0.05; OTH: 0.32 ± 0.07 mg/dL) and increase in creatinine clearance when compared with the OS group (OS: 6.0 ± 1.2; OSH: 10.2 ± 3.4; OT: 8.54 ± 2.53; OTH: 11.1 ± 2.8 mL/min/kg) (Figure 3A,B). There was no difference in plasma urea and proteinuria between the studied groups (Table 1).

**TABLE 1:** Renal function in the studied groups.

	OS	OSH	OT	OTH
Plasma urea (mg/dL)	33.8 ± 5.9	35.7 ± 4.8	32.3 ± 10.2	30.8 ± 5.1
Proteinuria (mg/24 h)	22.9 ± 4.3	19.1 ± 4.6	18.0 ± 3.3	20.1 ± 3.6

*Note*: Data are presented as mean ± standard deviation.

Abbreviations: OS, ovariectomized sedentary; OSH, ovariectomized sedentary treated with hydrochlorothiazide; OT, ovariectomized trained; OTH, ovariectomized trained treated with hydrochlorothiazide.

### 3.4 Renal morphology and morphometry assessments

**FIGURE 4:**
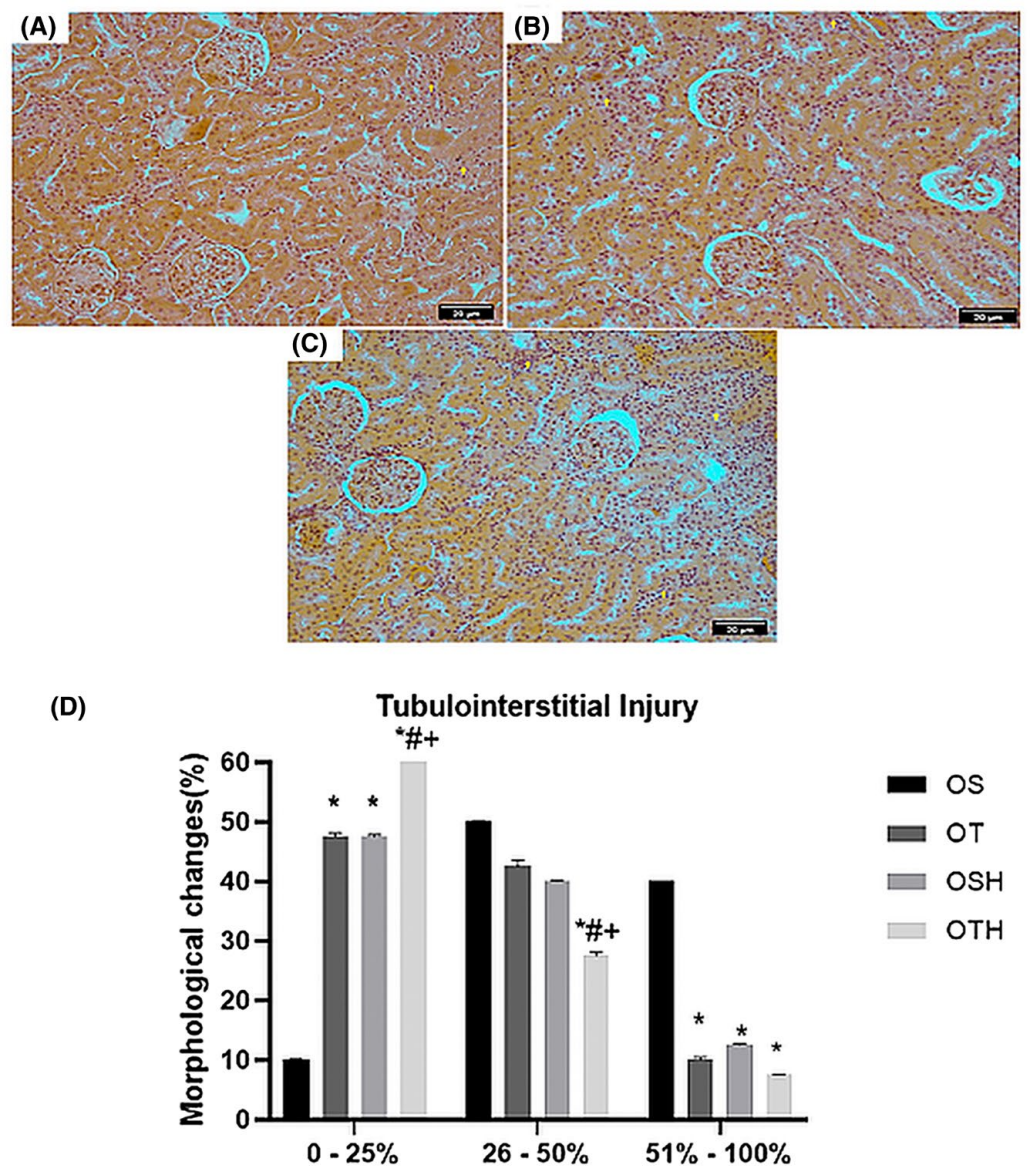
Representative histological sections of tubulointerstitial fibrosis stained with Masson's trichrome at 20× objective lens magnification. (A) tubulointerstitial fibrosis 0%–25%; (B) tubulointerstitial fibrosis 26%–50%; (C) tubulointerstitial fibrosis 51%–100%. + tubulointerstitial fibrosis. (D) Percentage of fields per band of interstitial tubulofibrosis in the studied groups. OS, ovariectomized sedentary; OSH, ovariectomized sedentary treated with hydrochlorothiazide; OT, ovariectomized trained; OTH, ovariectomized trained treated with hydrochlorothiazide. *p < 0.05 versus OS; ^#^ *p* < 0.05 versus OSH; ^+^ *p* < 0.05 versus OT.

For the analysis of tubulointerstitial fibrosis, the fields were divided into 3 ranges considering the morphological alteration: 0%–25%, 26%–50%, and 51%–100% (Figure 4A–C). The results demonstrated that the OS group presented a reduction in fields with a tubulointerstitial fibrosis area of 0%–25% and an increase in fields of 51%–100% when compared to the other studied groups. There was no difference between the OT and OSH groups in the analyzed fields of 0%–25% and 26%–50% of areas with interstitial tubulofibrosis. The OTH group showed an increase in fields with changes of 0%–25% and a reduction in the number of fields in the range of 26%–50% when compared to all other groups studied (Figure 4D).

### 3.5 Renal inflammation and oxidative stress

Regarding inflammatory mediators in renal tissue, the trained groups (OT and OTH) exhibited significantly reduced IL‐6 levels (OT: 240.22 ± 19.63 pg/mL and OTH: 259.75 ± 11.35 pg/mL) compared to the sedentary groups (OS: 321.90 ± 23.21 pg/mL and OSH: 274.15 ± 27.31 pg/mL). Additionally, the OTH group presented higher levels of IL‐10 (OTH: 100.26 ± 14.96 pg/mL) compared to the other groups in the study (OS: 87.96 ± 5.42 pg/mL, OSH: 76.66 ± 10.36 pg/mL, and OT: 76.93 ± 8.59 pg/mL). On the other hand, no statistically significant differences were observed between the groups regarding TNF‐α levels. (Table 2).

**TABLE 2:** Inflammatory mediators and markers of oxidative stress in kidney tissue in the studied groups.

	OS	OSH	OT	OTH
Inflammation				
TNF‐α (pg/mL)	93.24 ± 12.17	89.24 ± 13.72	99.46 ± 20.66	103.11 ± 3.91
IL‐6 (pg/mL)	321.90 ± 23.21	274.15 ± 27.31*	240.22 ± 19.63*^#^	259.75 ± 11.35*
IL‐10 (pg/mL)	87.96 ± 5.42	76.66 ± 10.36	76.93 ± 8.59*	100.26 ± 14.96*^#+^
Oxidative stress				
TBARS (μmol/mg protein)	1.87 ± 0.63	1.14 ± 0.33	1.15 ± 0.30	1.43 ± 0.36
NADPH oxidase (nmol/mg protein)	0.18 ± 0.03	0.16 ± 0.04	0.18 ± 0.04	0.18 ± 0.02
Hydrogen peroxide (μM)	3.70 ± 0.19	1.95 ± 0.46	1.61 ± 0.65	2.14 ± 0.39
Catalase (nmol/mg protein)	2.16 ± 0.30	3.50 ± 0.81	3.71 ± 0.49	3.87 ± 0.69*
Superoxide dismutase (USOD/mg protein)	6.99 ± 0.25	9.73 ± 1.18	9.79 ± 0.90	8.92 ± 1.46

*Note*: Data are presented as mean ± standard deviation.

Abbreviations: OS, ovariectomized sedentary; OSH, ovariectomized sedentary treated with hydrochlorothiazide; OT, ovariectomized trained; OTH, ovariectomized trained treated with hydrochlorothiazide.

*p < 0.05 versus OS; ^#^ *p* < 0.05 versus OSH; ^+^ *p* < 0.05 versus OT.

**FIGURE 5:**
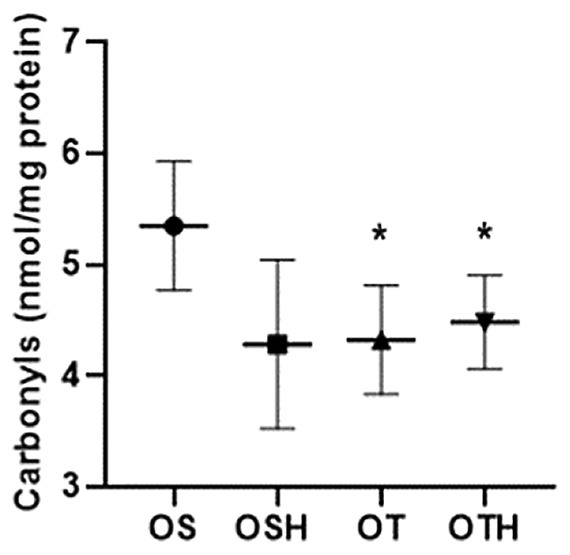
Protein oxidation (carbonyls) in renal tissue in the studied groups. OS, ovariectomized sedentary; OSH, ovariectomized sedentary treated with hydrochlorothiazide; OT, ovariectomized trained; OTH, ovariectomized trained treated with hydrochlorothiazide. *p < 0.05 versus OS; ^#^ *p* < 0.05 versus OSH; ^+^ *p* < 0.05 versus OT.

In the analysis of renal oxidative stress, both trained groups showed a reduction in protein oxidation assessed by carbonyls (OS: 5.35 ± 0.58 and OSH: 4.28 ± 0.76 vs. OT: 4.32 ± 0.49 and OTH: 4.48 ± 0.42 pg/mL) (Figure 5), and only the combination of drug and CET (OTH) promoted an increase in CAT activity when compared to the OS group (Table 2). There were no statistical differences between groups for TBARS, NADPH oxidase, hydrogen peroxide, and SOD in renal tissue.

### 3.6 Correlation

Positive correlations were obtained between variance of SBP and SBP (*r* = 0.72, *p* = 0.0003), plasma creatinine (*r* = 0.58, *p* = 0.008), IL‐6 (*r* = 0.62, *p* = 0.001), hydrogen peroxide (*r* = 0.61, *p* = 0.004), and protein oxidation (carbonyls) (*r* = 0.53, *p* = 0.010) in kidney tissue. Furthermore, increased vascular sympathetic modulation showed positive relationship with TBARS levels (*r* = 0.62, *p* = 0.001), suggesting that greater vascular sympathetic modulation favors greater lipoperoxidation.

## 4 DISCUSSION

Thiazide diuretics, such as HCTZ, are well‐established for controlling BP.^2^ However, the absence of positive modulation on different components and systems involved in the pathophysiology of the disease constitutes a remaining risk of the intervention. In this study, ovariectomized rats treated with HCTZ exhibited reduced SBP and improvements in renal morphology and function, including increased creatinine clearance and a reduction in the areas of moderate to severe tubulointerstitial fibrosis. Importantly, the combination of HCTZ and CET prevented an increase in SBP, as well as decreased variance of SBP and improved vascular sympathetic modulation. These results suggest that combined both pharmacological and non‐pharmacological approaches contribute to the observed reduction in SBP over the 8‐week.

Clinically, exercise training promotes multiple benefits for patients with CKD, including reduced BP, increased exercise tolerance, and pain relief, resulting in an improved quality of life.^31^, ^32^ In line with these findings, our experimental results in SHR with considerable degree of kidney injury show that the trained groups (OT and OTH) presented an increase in their performance when compared with the sedentary group (OS) in treadmill tests. Of note, OTH group, which combined HCTZ with CET, showed additional improvements in a maximal load test on stairs.

Thiazide diuretics and several classes of medications recommended for the treatment of SAH have efficient to reduce BP, corroborating with our findings. Nevertheless, our data demonstrate that the OTH group, combining HCTZ and CET, achieved the most significant reduction in SBP variability and marked improvements in sympathetic vascular modulation, emphasizing the substantial benefits of exercise training in improving the underlying mechanisms associated with development and progression of the SAH. The long‐term knowledge of the additional beneficial adaptations promoted by the inclusion of exercise training in antihypertensive pharmacological treatment has direct implications for the morbidity of individuals with SAH, although there is a lack of robust evidence on the subject. In this regard, the experimental findings of our study open perspectives for new investigations, including the use of other pharmacological approaches and longitudinal study with hypertensive patients.

The relationship between sympathetic hyperactivity and SAH is well‐documented. Grassi et al. reported an increase in muscle sympathetic nervous activity with the progressive increase in BP in pre‐hypertensive and hypertensive individuals.^33^ Also, increased BPV is also associated with higher mortality risks and damage to organs, such as the kidneys.^34^ It is worth remembering that cardiovascular autonomic dysfunction, characterized by sympathetic hyperactivity, can impair baroreceptor sensitivity and increase BPV, which plays a critical role in renal damage.^35^ Moreover, autonomic dysfunction has been shown to modulate inflammation and oxidative stress, exacerbating renal injury.^20^, ^35^ In the present study, we observed that only the combination of HCTZ and CET (OTH group) reduced sympathetic modulation in SHR subjected to ovarian hormone deprivation, which may be involved in the reduction of SBP and improvement in renal function. Previous studies have shown that HCTZ, in doses similar to those used here, improves baroreflex sensitivity and reduces SBP variability in male SHR, resulting in less organ damage, including to the kidneys.^36^, ^37^ Here, the BP reduction and the morphological improvement after treatment with HCTZ (OSH group) do not appear to be explained, even partially, by the SBP variability, suggesting that other mechanisms may have predominantly contributed to the maintenance of BP at elevated levels.

Several factors contribute to renal damage, including dysregulation of the renin‐angiotensin‐aldosterone system (RAAS), sympathetic nervous system hyperactivity, inflammation, and oxidative stress.^38^, ^39^ Importantly, the dysregulation of sodium homeostasis and, consequently in volume control, as well as RAAS, are influenced by inflammatory cytokines and reactive oxygen species (ROS).^38^, ^39^ Regarding autonomic mechanisms, reduced baroreflex sensitivity, increased sympathetic nervous system activity, and BPV have been associated with increased inflammation and oxidative stress, collectively contributing to renal morphological and functional alterations and CKD.^40^ In turn, increased sympathetic tone also leads to changes in renal hemodynamics, contributing to tissue damage and inflammatory responses, which aggravates SAH and promotes kidney injury.^3^, ^40^

In the present study, we found that the trained groups showed reduced IL‐6 levels, with the OTH group displaying a significant increase in IL‐10 compared to other groups. This suggests that combining HCTZ with CET enhances anti‐inflammatory responses in renal tissue. Additionally, oxidative stress parameters showed that both trained groups (OT and OTH) had lower levels of protein oxidation (carbonyls) in renal tissue. Moreover, only the OTH group demonstrated a significant increase in CAT activity (vs. OS group), indicating enhanced antioxidant defenses. Our data are consistent with previous studies in which exercise training contributes to reducing renal oxidative stress.^41^, ^42^ Furthermore, in a recent study from our research group, we observed that both types of training (aerobic and resistance exercises) were effective in reducing renal oxidative stress and improvement autonomic control of circulation in ovariectomized rats with metabolic syndrome.^42^

Our findings are in line with the idea that reduced renal sympathetic nerve activity improves kidney function, as evidenced by increased creatinine clearance and improved kidney morphology.^43^ Here, all intervention groups (OSH, OT, and OTH) showed reduced tubulointerstitial fibrosis compared to the OS group, with the OTH group demonstrating the most significant benefits, including an increase in areas with mild fibrosis (range of 0%–25%) and a reduction in moderate fibrosis (range of 26%–50%).

The improvement in BPV and the reduced sympathetic vascular modulation associated with decreased inflammation suggest the involvement of the anti‐inflammatory cholinergic reflex. It has been demonstrated the interaction between sympathetic hyperactivity, oxidative stress, and kidney injury, highlighting the therapeutic potential of modulating these pathways to improve outcomes in SAH.^44^ In fact, we observed that elevated BPV is associated with markers of inflammation, such as IL‐6, and oxidative stress, including protein oxidation. Furthermore, we obtained positive correlations between LF‐SBP and lipoperoxidation. Grassi et al.^45^ highlighted that sympathetic overactivity and impaired baroreflex sensitivity significantly contribute to cardiovascular complications, particularly in hypertension‐related left ventricular dysfunction. This study reinforces the link between autonomic imbalance and end‐organ damage, further emphasizing the importance of strategies targeting sympathetic modulation. The LF‐SBP has been shown to be a robust predictor of cardiovascular risk in hypertensive patients, with increased LF‐SBP associated with higher cardiovascular morbidity. These findings align with our observations and emphasize the need for interventions to modulate BPV and sympathetic activity.^46^ Furthermore, the combination of pharmacological and non‐pharmacological interventions, such as HCTZ and CET, appears to enhance autonomic balance, reduce BPV, and improve renal outcomes, likely mediated by anti‐inflammatory and antioxidant mechanisms.

A key observation in our study was the positive correlation between variance of SBP and markers of inflammation (IL‐6) and oxidative stress (hydrogen peroxide and protein oxidation). In addition, vascular sympathetic modulation, represented by LF‐SBP, and lipoperoxidation, represented by TBARS levels, showed a positive relationship. These findings suggest that BPV, inflammation, and oxidative stress are interconnected, and that reducing BPV through combined therapy improves kidney function by modulating these factors. Notably, the OTH group showed the most favorable redox profile, which may have contributed to the observed improvement in renal function.

The lack of normotensive control and/or non‐ovariectomized groups may be seen as a limitation of our experimental design. However, the detrimental effects of SAH and ovarian hormone deprivation are well‐documented in the literature. Additionally, this study was designed to examine the potential benefits of combining pharmacological and non‐pharmacological treatments on BPV and renal parameters in a model of SAH with ovarian hormone deprivation. In this context, the experimental design of this study employs a sedentary hypertensive group subjected to ovariectomy as a control group, allowing us to make the mentioned comparisons in alignment with the proposed objectives. Thus, we believe that additional control groups would not substantially alter the conclusions drawn from our data. We also emphasize that the early deprivation of ovarian hormone in adult rats is justified by the confounding factor of aging. Data obtained from rats undergoing ovarian hormone deprivation later in life (i.e., at more advanced ages) do not allow us to accurately determine whether the findings are a result of ovarian hormone deprivation or the aging process itself. In this sense, the findings of the current study may differ from those observed in elderly rats.

Based on our experiment, our study suggests that the combination of HCTZ and CET provides superior benefits compared to HCTZ alone in female SHR subjected to ovarian hormone deprivation. This combined therapy significantly reduced BPV, improved inflammatory and redox profiles, and provided functional and morphological benefits to renal tissue. From a translational perspective, replicating similar results in humans would highlight the fundamental importance of including regular exercise training in antihypertensive pharmacological treatment to reduce cardiovascular and renal morbidity in postmenopausal women, providing additional benefits beyond BP reduction and potentially leading to an improved quality of life and reduced hypertension‐related complications.

## AUTHOR CONTRIBUTIONS

P. P. Neves, M. J. Ferreira, G. N. Gomes, and K. De Angelis conceived and designed the research; P. P. Neves, M. J. Ferreira, T. P. Shecaira, and M. R. H. Dutra performed the research and acquired the data; P. P. Neves, M. R. H. Dutra, D. C. Kimura, G. N. Gomes, and K. De Angelis analysed and interpreted the data. All authors were involved in drafting and revising the manuscript.

## FUNDING INFORMATION

This study was supported by the Coordination for the Improvement of Higher Education Personnel (CAPES), Universidade Nove de Julho (UNINOVE), São Paulo Research Foundation (FAPESP 2019/06277–0), and National Council for Scientific and Technological Development (CNPq: 151838/2024‐0, 407398/2021–0, and 406792/2022–4). M.J.F. (CNPq ‐ PDJ) and K.D.A (CNPq ‐ BPQ) are recipients of CNPq Fellowship.

## CONFLICT OF INTEREST STATEMENT

The authors declare that they have no known competing financial interests or personal relationships that could have appeared to influence the work reported in this manuscript.

## ETHICS STATEMENT

All applicable international, national, and/or institutional guidelines for the care and use of animals were followed. Ethical approval for this study was obtained from the Ethics Committee for Ethics in the Use of Animals—CEUA of UNIFESP (approval number: 7611290618). In addition, the authors confirm that all methods were performed in accordance with the relevant guidelines and regulations.

## ARRIVE GUIDELINES

The authors confirm that the study is reported in accordance with ARRIVE guidelines.

## Data Availability

The data that support the findings of this study are available from the corresponding author upon reasonable request.
